# Periprostatic Adipose Tissue: A New Perspective for Diagnosing and Treating Prostate Cancer

**DOI:** 10.7150/jca.89750

**Published:** 2024-01-01

**Authors:** Hongliang Cao, Yishu Wang, Difei Zhang, Bin Liu, Honglan Zhou, Song Wang

**Affiliations:** 1 Department of Urology II, The First Hospital of Jilin University, Changchun 130021, China.; 2 Key Laboratory of Pathobiology, Ministry of Education, Jilin University, Changchun 130021, China.

**Keywords:** prostate cancer, periprostatic adipose tissue, lipids, inflammation, diagnosis, and treatment

## Abstract

Prostate cancer (PCa) is the most common tumor of the male genitourinary system. It will eventually progress to fatal metastatic castration-resistant prostate cancer, for which treatment options are limited. Adipose tissues are distributed in various parts of the body. They have different morphological structures and functional characteristics and are associated with the development of various tumors. Periprostatic adipose tissue (PPAT) is the closest white visceral adipose tissue to the prostate and is part of the PCa tumor microenvironment. Studies have shown that PPAT is involved in PCa development, progression, invasion, and metastasis through the secretion of multiple active molecules. Factors such as obesity, diet, exercise, and organochlorine pesticides can affect the development of PCa indirectly or directly through PPAT. Based on the mechanism of PPAT's involvement in regulating PCa, this review summarized various diagnostic and therapeutic approaches for PCa with potential applications to assess the progression of patients' disease and improve clinical outcomes.

## 1. Introduction

Prostate cancer (PCa) is the most common malignancy of the male genitourinary system. Among men in the United States, the estimated number of new PCa cases in 2023 ranks first among all tumors and second in mortality, second only to lung cancer, with an increasing trend every year 1. More than 80% of patients with PCa are diagnosed with localized or locally advanced PCa. This stage can be treated using active surveillance, radical surgery, or radiotherapy alone or combined with androgen deprivation therapy (ADT), with satisfactory clinical outcomes 2. Endocrine therapy is an essential treatment for patients with advanced PCa. However, after a median duration of 18‒24 months of endocrine therapy, PCa progresses to metastatic castration-resistant prostate cancer (mCRPC), defined as PCa that has reached harmful levels of serum testosterone (<50 ng/dl or 1.7 nmol/L) after an initial continuous ADT therapy, persistently elevated prostate-specific antigen (PSA) levels or imaging on progression, reduced patients' quality of life, and shorter survival 3, 4. The standard first-line treatment chemotherapy drug for treating mCRPC is docetaxel-based drugs 5. Second-generation androgen receptor (AR) signaling inhibitors (e.g., enzalutamide 6) and intratumoral androgen synthesis inhibitors (e.g., abiraterone 7) have been testified for treating mCRPC and have improved survival benefits; however, the prognosis of mCRPC remains poor. Therefore, there is an urgent need for new diagnostic and therapeutic tools for PCa.

Adipose tissues are divided into visceral and subcutaneous adipose tissues according to their anatomical location. Other adipose tissues are in the perivascular, bone marrow cavity, and ectopic storage (e.g., nonalcoholic fatty liver and pancreatic adipose tissue accumulation). Visceral adipose tissues are primarily distributed around the mesentery and omentum 8, 9. Adipose tissues are classified into white adipose tissues (WAT) and brown adipose tissues (BAT) based on morphological and functional characteristics. WAT is the primary source of physiological fuel and consists of monocular lipid droplets that constitute 95% of adipocytes, a non-thermal energy-storing adipocyte that also provides mechanical protection and resistance to infection and injury 10. BAT has a limited distribution and is only found in the neck, shoulders, posterior thorax, and some anatomical depots in the abdomen, accounting for 0.2 to 3.0% of total adipose tissue mass 11. It consists of multicompartmental lipid droplets dispersed in the mitochondria-rich cytoplasm and mediates thermogenesis mainly via uncoupling protein 1 (UCP-1) pathway, and some other thermogenic mechanisms have been shown to exist, based on ATP sinks centered on creatine, lipid, or calcium cycling, along with Fatty acid-mediated UCP1-independent leak pathways driven by the ADP/ATP carrier (AAC) 12. Adipose tissues consist of many adipocytes, other non-adipocytes, connective tissue matrix, blood vessels, and nerve tissues. The non-adipocyte components include inflammatory cells (macrophages), immune cells, preadipocytes, and fibroblasts 13. These components, as a whole, affect body lipid metabolism, insulin sensitivity, inflammation, energy homeostasis, angiogenesis, and cell proliferation 14. Adipose tissue activity is associated with the development of various tumors 15, 16. Different adipose depots have different morphological and functional characteristics and have different effects on different tumors 17, 18.

Periprostatic adipose tissues (PPAT) are located in the pelvic region and are largely surrounded by a prostatic envelope separated by a layer of fibromuscular sheets of varying thickness, crossed by prostatic vessels and are the closest adipose tissues to PCa. Forty-eight percent of the prostate surface has PPAT, and 44%, 36%, 59%, and 57% of the anterior, posterior, right, and left surfaces have adipose tissue distribution, respectively. Besides, one third of the anterior prostate is in direct contact with PPAT 19, 20. PPAT is generally considered a white visceral adipose tissue. However, in some cases, Alvarez-Artime, A. et al. speculated that PPAT could be transformed into a beige adipose tissue, possessing white and brown adipose tissue characteristics 21. Compared with subcutaneous adipose tissues, PPAT has unique morphological and functional characteristics; the adipocytes in PPAT are smaller, have the same basal rate of fatty acid release (lipolysis) but release fewer types of polyunsaturated fatty acids and are more sensitive to isoprenaline-stimulated lipolysis 22. Therefore, they play a different role than other adipose tissues and their study alone has some significance. PPAT affects various prostate-related diseases, such as prostatitis, benign prostatic hyperplasia (BPH), secondary lower urinary tract symptoms, erectile dysfunction, urethral dysfunction, and PCa 23, 24, 25. PPAT is an active secretory organ that can affect the PCa lipid microenvironment and inflammatory state, thus promoting PCa progression by secreting lipids, adipokines, and hormones in a paracrine or endocrine manner. PPAT also directly contacts PCa or mediate communication between PPAT and PCa in an exocytic manner 26, 27. In turn, PCa regulates the biological behavior of adipose tissues, thus promoting its development 26, 28, 29. Only a few studiies have examined the effects of PCa on PPAT. Hence, to clearly describe the role of PPAT on PCa, this article mainly described the unidirectional effects of PPAT on PCa.

## 2. PPAT Regulates the Lipid Metabolism of PCa and Changes the Tumor Lipid Microenvironment

Unlike most tumors, early PCa adapts to the energy required for tumor survival and proliferation mainly through lipid metabolic reprogramming of fatty acid β-oxidation for energy supply. As the tumor progresses, glycolysis is gradually enhanced and cancer cells gradually show the Warburg effect with a higher rate of glucose uptake 30. The process of lipid metabolic reprogramming plays a role in some researchs. Gazi et al. found that lipid-specific translocation between adipocytes and PCa cells by utilizing labeled fatty acids, which appears by direct cellular contact or paracrine 31. PPAT explants from post-radical PCa co-cultured with PCa cell lines showed decreased expression of lipid metabolism genes (CD36, FASN, PPARG, and CPT1A), indicating a progressive decline in PPAT lipid production and utilization, contrary to that discovered in co-cultured PCa cell lines. Increased lipid absorption and accumulation in PCa cells and increased number of intracellular lipid droplets were associated with increased PCa aggressiveness. They inhibited PCa growth in vivo and in vitro 32, 33. These studies suggest that PPAT might be a significant source of fatty acids for PCa cells. The metabolic processes and metabolism-related proteins of free fatty acids (FFAs) in PCa correlate with the biological behavior of PCa. PCa cells take up lipids mainly utilizing macrocytic drinking or fatty acid transporter protein CD36 and store them in the cyto-plasm as lipid droplets (LDs). Targeting CD36 reduces FFAs uptake and slow cancer progression 34. Fatty acid-binding proteins (FABPs) are a family of proteins that serve as intracellular FFAs transporters and are related to the intracellular storage of FFAs, which are translocated to the nucleus in PCa cells to interact with peroxisome proliferation-activated receptor γ (PPARγ) and promote cell proliferation, invasion, and migration 35. In addition to classical cytoplasmic lipolysis by lipases, lipid droplets also release FFAs through lipophagy, which then provides energy for β-oxidation in the mitochondria. Lipophagy, a selective form of autophagy, is associated with LD degradation. In locally progressive PCa, cancer cells have elevated levels of lipid droplets and autophagy markers in extraprostatic regions in contact with PPAT, and these markers correlate with PCa aggressiveness 36. These experiments suggest that FFAs secreted by PPAT influence the reprogramming of PCa lipid metabolism and, thus, its progression.

In addition, the amount and type of lipids released by PPAT indicate the risk of PCa progression, and current studies have focused on FFAs. FFAs are classified into saturated fatty acids (SFAs) and unsaturated fatty acids (UFAs) based on the number of double bonds, the latter including monounsaturated fatty acids, polyunsaturated fatty acids (PUFAs), and n-3 and n-6 PUFAs. The fatty acid composition of PPAT as determined using in vitro magnetic resonance (MR) spectroscopy by Iordanescu et al. suggested that the FFA composition of PPAT was changed in patients with aggressive PCa. The unsaturated to saturated fatty acid ratio showed a moderate negative correlation with the Gleason score 37, 38. Similarly, Altuna-Coy et al.'s study suggested that lipidomic and functional analyses of PPAT indicated lipidomic differences between low and high-risk PCa, with alterations in fatty acid biosynthesis, linoleic acid metabolism, and β-oxidation of very long chain fatty acids having the most significant impact on the PPAT lipidome. When PPAT was grouped according to risk, palmitic, stearic, arachidonic, docosanoic, and linoleic ac-ids (LA) and their metabolites showed a trend toward reduction 33. Figiel et al. suggested that PUFA composition in PPAT reflects past PUFA absorption, is related to PCa aggres-siveness, and varies according to geographic origin. Low levels of n-6 PUFAs, such as Lin-oleic acid, and high levels of SFAs were associated with PCa aggressiveness in African-Caribbean patients, and n-6 PUFAs were twice as high as in Caucasian patients. Low lev-els of n-3 PUFAs, such as eicosapentaenoic acid (EPA), were associated with PCa invasiveness in Caucasian patients. The in vitro migration potential of PPAT FFA extract-supplemented PCa cell lines was negatively correlated with adipose tissue LA content 39. Interestingly, a study analyzing the basal secretory FA profile of PPAT exosomes showed no difference between patients with weaker or stronger PCa according to the Gleason score and tumor aggressiveness, and they concluded that there was no relationship between altered biological behavior of PCa and PPAT metabolic reprogramming in obese men 22. In addition, studies have suggested that cholesterol metabolism in PPAT is also altered, with African-Caribbean patients having lower levels of cholesterol esters in PPAT than Caucasian patients, without any association with markers of PCa aggressiveness. In PCa tissues from African-Caribbean patients, the amount of ABCA1 (aasociated with cholesterol efflux) was reduced and the expression of SREBP-2 (associated with cholesterol uptake) was increased, and the direction of cholesterol accu-mulation in cancer cells correlated with a more frequent epithelial-mesenchymal transition (EMT) status, which may promote PCa aggressiveness in this way 40. These findings demonstrate that PPAT alters the lipid composition of the PCa microenvironment, which in turn affects PCa progression.

The specific mechanisms for the action of FFAs on PCa in the PPAT microenvironment have been reported. EPA regulates protein kinase C signaling pathway and Akt kinase activity in PCa cells and suppress the growth of PCa xenografts 41, 42. Figiel et al. found that in the EMT, transcription factor Zeb1 and the Ca2+-activated positive feedback loop between the K+ channel SK3 amplified Ca2+ entry and cell migration. In vitro experiments using human PCa sections and in vitro cultures found that LA and EPA exert anti-cancer effects by regulating Ca2+ entry, which is involved in Zeb1 regulation and cancer cell migration 43. PPAT co-cultured with PCa or exogenous FFAs induces the expression of NOX5, an isoform of NADPH oxidase, which increases intracellular reactive oxygen species (ROS) and activates the HIF1/MMP14 pathway, which increases tumor cell invasion. In obese patients/samples, adipocytes surrounding the tumor are more likely to activate the described signaling pathway and induce tumor invasion 44. Adipocytes in PPAT can also directly stimulate PC3 cells to produce MIC-1 (TGF-β family) and prostate mesenchymal fibroblasts to secrete IL-8 by upregulating lipolysis and FFA release. MIC-1 is a TGF-β family molecule, and the enhanced overexpression and secretion of MIC-1 stimulates PCa cell proliferation and invasion and are involved in anticancer therapy resistance 45. All these mechanisms suggest that PPAT regulates PCa development through the release of FFAs.

## 3. Factors Secreted by PPAT Impact PCa

PPAT is an active secretory organ that secretes various factors which regulates multiple biological PCa behaviors, including cell proliferation, migration, and invasive capacity 46, which is currently a topic of interest in adipose tissue research. The current factors involved in PPAT secretomes are FFAs, leptin, lipocalin, interleukins, TNFα, chemokines, growth factors, and androgens. These molecules have highly diverse chemical structures and physiological functions, and the effect of PPAT on PCa cells depends on the balance between the pro- and anti-cancer effects of these molecules, which deserves further investigation. We will next discuss pilot studies confirming these molecules. Finley et al. found that interleukin-6 (IL-6) levels in PPAT CM (conditioned medium) was approximately 375-fold higher than that in patient-matched serum, correlated with pathological grade, and IL-6-regulated Stat3 phosphorylation levels were higher in high-grade tumors. This suggests that PPAT may regulate the aggressiveness of PCa by acting as a source of IL-6 47. In addition, transgenic expression of IL-6 in the mouse prostate induced autocrine IL-6 and homeostatic activation of STAT3 in prostate tissues, upregulated insulin-like growth factor (IGF) paracrine secretion, reprogrammed prostate oncogene expression, induced PCa production, and amplified inflammation in the prostate and PPAT 48. Upregulated IL-6 in PPAT may also induce the development of hormone-refractory PCa by promoting neuroendocrine differentiation, inducing androgen production in the prostate, and activating androgen receptors 49. Zhang et al. also demonstrated that IL-6 was highly expressed in PPAT, and lipocalin was lowly expressed. In addition, IL-6, leptin, and creactive protein levels are significantly elevated with increased PCa aggressiveness, and PPAT quantity increased significantly 50, 51.

In addition to IL-6, matrix metalloproteinases (MMPs) and chemokines play essential roles in the PPAT microenvironment; they promote PCa invasion and metastasis. Extracellular matrix metalloproteinases play significant roles in basement membrane and extracellular matrix degradation, thus promoting tumor invasion and metastasis. Therefore, they are of great interest in cancer research 52. Sacca et al. demonstrated that PPAT CM secretes more pro-MMP-9 than BPH CM, promoting the invasive ability of PCa 53. Ribeiro et al. observed increased MMP2 and MMP9 activity in PPAT and increased proliferation and migration capacity when PC-3 cells were stimulated with PPAT CM 54. The analysis of the stromal vascular fraction (SVF) of PPAT in 6-month-old obese HiMyc mice by Saha et al. suggested that the levels of SVF encoding various chemokines, cytokines, and mRNAs encoding various chemokines, cytokines, growth factors, and angiogenic mediators were significantly increased, CXCL12 gene being one of the most significantly upregulated genes. CXCL12 receptors CXCR4 and CXCR7 were expressed in PCa cell lines and HMVP2 cells, and CXCL12 stimulated the migration and invasion of HMVP2 cells but not control cells. The effect of CXCL12 on HMVP2 cells were inhibited by the CXCR4 antagonist AMD3100 and by the knockdown of CXCR4 or CXCR7. CXCL12 treatment also rapidly activated STAT3, NFkB, and MAPK signaling in HMVP2 cells, which were again attenuated by AMD3100 or CXCR4, or CXCR7 knockdown 55. Another study showed that PPAT secretes the chemokine CCL7, which diffuses from PPAT into the peripheral zone of the prostate and stimulates the migration of CCR3-expressing tumor cells. When UCB35625 inhibited the CCR3/CCL7 axis, the observed increase in migration associated with obesity completely disappeared 56.

In addition, the role of factors such as TNF-α, VEGF, TGF-β, IGF-1, and androgens in the PPAT microenvironment has been demonstrated. Dahran et al. demonstrated that the expression levels of TNF-α and VEGF on immunostaining in radical prostatectomy (RP) resected PPAT correlated significantly with the aggressiveness of PCa, suggesting the risk of higher-grade PCa 57. Civita et al. found that PPAT CM culture promoted the migration of two different human androgen non-dependent (AI) PCa cell lines (DU145 and PC3) and upregulated CTGF expression. The well-known TGF-β receptor inhibitor SB431542 counteracted the increased migration and reduced CTGF expression observed in the presence of AdipoCM, suggesting that paracrine secretion of TGF-β by PPAT affects PCa cell motility 58. Liotta et al. also demonstrated that PPAT upregulates TUBB2Bβ-microtubulin by paracrine. Moreover, IGF-1 isoform promotes resistance to docetaxel in PCa, an effect partially counteracted by the IGF-1 receptor inhibitor AG1024 59. Another study investigated all steroid hormones, including active androgens, in human PPAT tissues utilizing liquid chromatography-tandem mass spectrometry (LC-MS/MS). Steroid hormones, including active androgens and androgen synthase CYP17, CYP19, and 5-α-reductase activity, were confirmed in human adipose tissues and may be associated with CRPC through the stimulation of androgen receptor cancer cell development 60, 61. In addition to the above experimentally confirmed factors, AlZaim, I. et al. also speculated that PPAT may act on PCa through visfatin, omentin,resistin, LCN2, RBP4, osteopontin, chemerin, apelin and other factors, but this needs further verification 62.

Another study using LC-MS/MS-based proteomic analysis revealed the proteomics in PPAT. Compared with CM-BPH, proteins that involved in different biological processes of PCa were expressed diversely. For example, proteins about locomotion, reproduction, immune system functions, catalytic activity, defense activity, transport proteins, metabolism and energy pathways expressed differentially in both groups 63. These results revealed that multiple differentially expressed proteins in PPAT influence PCa, which warrants further investigation.

In addition to the factors that PPAT can secrete to regulate PCa, PCa can also alter PPAT function, thus promoting its development. The stimulation of PPAT exosomes by PC-3 CM induced the secretion of the bone-bridging protein, TNF-α, and IL-6, which are associated with cancer progression, upregulation of bone-bridging protein expression by 13-fold, and decreased expression of the protective adipokine lipocalin. The stimulation of matrix metalloproteinase-9 activity and higher mitochondrial DNA copy number suggests that PPAT plays a vital role in PCa progression^29^. Vitamin D receptor deficiency in mice with PCa induces fat necrosis and individual cell apoptosis in PPAT, which regulates PCa signaling pathways and affects PCa progression 64.

## 4. Inflammation of PPAT Influences the Progression of PCa

During weight gain, adipocytes accumulate lipids, become hypertrophic, hypoxic, and eventually their cells die. This cycle increases adipocyte chemokine production and immune cell recruitment, ultimately triggering chronic white adipose tissue (WAT) inflammation associated with carcinogenesis by releasing pro-inflammatory cytokines from adipocytes and immune cells. The pathology of WAT inflammation is characterized by coronal structures (CLS) consisting of dead or dying adipocytes surrounded by macrophages. These macrophages remove lipids and cellular debris and sometimes evolve into a multinucleated giant or foam cells. CLS is associated with a worse prognosis in patients with cancer, and interest in using these structures as prognostic biomarkers is growing 65.

Chronic inflammation and CLS formation also occur in PPAT and are associated with PCa progression. In a prospective study of 169 men with newly diagnosed PCa, periprostatic WAT inflammation was found in 49.7% of patients. It was associated with higher body mass index (BMI), larger adipocyte size, and tumors with a Gleason classification of IV/V. The association between PPAT inflammation and high Gleason grade remained significant after adjustment for BMI 66. Polymerase chain reaction analysis of PPAT and subcutaneous adipose control tissues (SAT) collected from patients with PCa undergoing radical prostatectomy or BPH control patients undergoing simple prostatectomy showed that many inflammatory genes (e.g., IL8RA, ILRAB, CXCL2, CCL8, and CCL21) in PPAT compared with SAT were associated with high grade (Gleason-9) PCa. Moreover, CCL2, CCL4, and CXCL1-3 were downregulated 67. The mouse DIO model exhibits marked PPAT inflammation secondary to AT expansion, with increased expression of CD68, MCP1, and TNF-α. They increased CLS formation, consistent with the enrichment of inflammatory response pathways 68, 69. These studies all suggest an active inflammatory process in PPAT and is closely related to the development of PCa.

PPAT WAT inflammation may result from hypoxia and endoplasmic reticulum stress, and hypertrophic adipocytes that are hypoxic beyond the vascular support may be more sensitive to cell death. Endoplasmic reticulum stress in hypertrophic adipocytes leads to apoptosis, triggering an inflammatory response 70, 71. Inflammation in PPAT is also associated with higher insulin, triglyceride, and leptin/lipocalin ratios, and lower high-density lipoprotein cholesterol, adipocyte size, and PCa levels compared with pa-tients without PPAT inflammation. In contrast, hyperinsulinemia promotes PCa cell proliferation, inhibits apoptosis, and is associated with adjuvant steroidogenesis. This stimulates the prostate by activating androgen receptors adenoma formation and activating androgen receptors 66. AlZaim, I. et al. speculated that obesity, metabolic syndrome and diabetes may lead to PPAT inflammation and further affect PCa development by activating Thrombin cascade, while targeting Thrombin, Factor Xa, and protease-activated receptors (PARs) factors in the thrombin system may inhibit this process. In addition, caloric restriction, weight loss, and weight loss surgery, estrogen supplementation, and antidiabetic drugs can improve the efficacy of PCa treatment by improving the inflammatory state of PPAT 62.

## 5. Some Elements that Indirectly (through PPAT) or Directly Affect PCa

Obesity is a chronic increase in excess adipose tissues 17. The direct link between obesity and PCa remains controversial. Histological analysis of PCa after transplantation of patient-derived PCa grafts (PDXs) in lean or obese combined immunodeficient (SCID) mice in culture for 10 weeks suggested that systemic obesity did not promote prostate tumorigenesis, neither did the transplantation of PPAT and PDXs together enhance tumor-igenesis 72. In contrast, several studies have shown that obesity is associated with an increased risk of PCa and poor prognosis, which can be explained by elevated levels of se-rum adipokines such as IL-6, leptin, TNF-α, CCL7, CXCL12, CXCL-1, VEGF, MCP-1, in-creased MMP-9 activity, and altered metabolism of sex hormones in obese individuals 13, 15, 73. Despite the controversy, several studies have suggested an indirect mechanism of action of obesity through PPAT affecting PCa. Obesity can induce PPAT inflammation, as evidenced by high CLS density and elevated levels of pro-inflammatory mediators. PPAT inflammation is more common in overweight and obese men; however, it is also detected in more than 40% of men with BMI <25, and PPAT volume does not increase during obesity, which may be because of the chronic hypoxic state of PPAT causing inflammation and fibrosis limiting its expansion 71. Furthermore, during obesity, PPAT is more active in metabolism and secretion in obese men than in lean men, although PPAT volume does not increase. For example, obesity strongly promotes the process by which PPAT-secreting chemokine CCL7 stimulates the migration of CCR3-expressing tumor cells 56. The expression of NOX5 and MMP14 is upregulated at the front end of PCa invasion, a process that is amplified in patients with obesity 44. PPAT secretions obtained from patients with obesity stimulate PCa cell proliferation and angiogenesis more effectively than lean patients. The entire epigenomic methylation profile of PPAT was significantly different in obese or overweight patients compared with normal-weight patients with PCa. Epigenetic variants associated with excessive obesity may alter lipid metabolism and immune dysregulation, resulting in an unfavorable PCa microenvironment 74. PPAT from obese patients with PCa stimulates higher rates of PCa and endothelial cell proliferation compared with subcutaneous adipose tissues from lean or obese patients. In addition, obesity alters the fatty acid (FA) profile in PPA and increases angiogenesis 75. The function of prostates depends partially on direct hormone receptors on prostate epithelial cells and indirectly on systemic metabolism, including the effects of obesity 76. Saha, A. et al. reviewed the potential mechanisms by which PPAT promotes PCa in the course of obesity, with particular emphasis on the important role of adipose stromal cells (ASCs) 77.

Scheinberg et al. summarized the relationship between dietary fat intake and the risk of PCa and found contradicting results from various studies. Possible mechanisms by which fat intake increases the occurrence of PCa include the effects on hormone regulation and androgen levels, oxidative stress, inflammation, exposure to toxic pesticides, and the effects of specific fatty acids 78. Another review suggested that reducing red meat and saturated fat intake could prevent PCa. Consumption of n-3 and n-6 PUFA, phytoestrogens, and different dietary patterns affect the risk of PCa 79. All these factors can indirectly affect PPAT. The current findings are less uniform; however, a high-fat diet (HFD) can induce obesity to act on PPAT indirectly. Furthermore, a direct effect on PCa progression through blood circulation has been proposed in several studies. Bhardwaj et al. verified that HFD could induce PPAT inflammation in mice. In contrast, the restriction of calorie intake in obese mice resulted in weight loss, reduced PPAT inflammation, and reduced expression of pro-inflammatory genes. Further PPAT transcriptome analysis revealed that excessive calorie intake enriched the inflammatory response pathways, whereas the restriction of calorie intake normalized inflammatory response pathways 68.

Rocha-Rodrigues et al. proposed that exercise training reduces visceral fat volume, increases skeletal muscle mass in patients with PCa, and improves the tumor microenvironment. Exercise training influences PCa progression by modulating the secretion of adipokines from PPAT and other adipose tissues and the secretion of myokines from skeletal muscles 80. In addition, the participation of patients with PCa in exercise training after diagnosis may improve their survival 81. Moderate aerobic exercise in young people may decrease the circulating levels of free IGF-1 and reduce the potential to support PCa growth 82. The mechanisms by which physical activity affects PCa are complex and unknown; however, the potential of physical training to influence PCa progression through PPAT warrants further investigation.

Organochlorine pesticides (OCPs), a series of persistent organic pollutants with endocrine disrupting and bio-accumulative properties 83, are highly lipophilic and tend to accumulate in tissues with high-fat content. Recently, McKinlay et al. suggested that the exposure pattern of OCPs varies according to the ethnic-geographic origin, with most OCPs being present in higher concentrations in Caucasian patients. In addition, pp'-DDE (a kind of OCPs) levels are twice as high in African-Caribbean patients. Chlordecone is only detected in African-Caribbean patients with PPAT. Most OCP concentrations are positively correlated with age and BMI. After adjusting for age, BMI, and PUFA composition of PPAT, no significant correlation was found between OCP levels and the risk of aggressive diseases, except for mirex, which was negatively correlated with aggressive PCa characteristics in Caucasian patients 83.

## 6. PPAT Provides New Perspectives for Diagnosing and Treating Prostate Cancer

**Figure 1:**
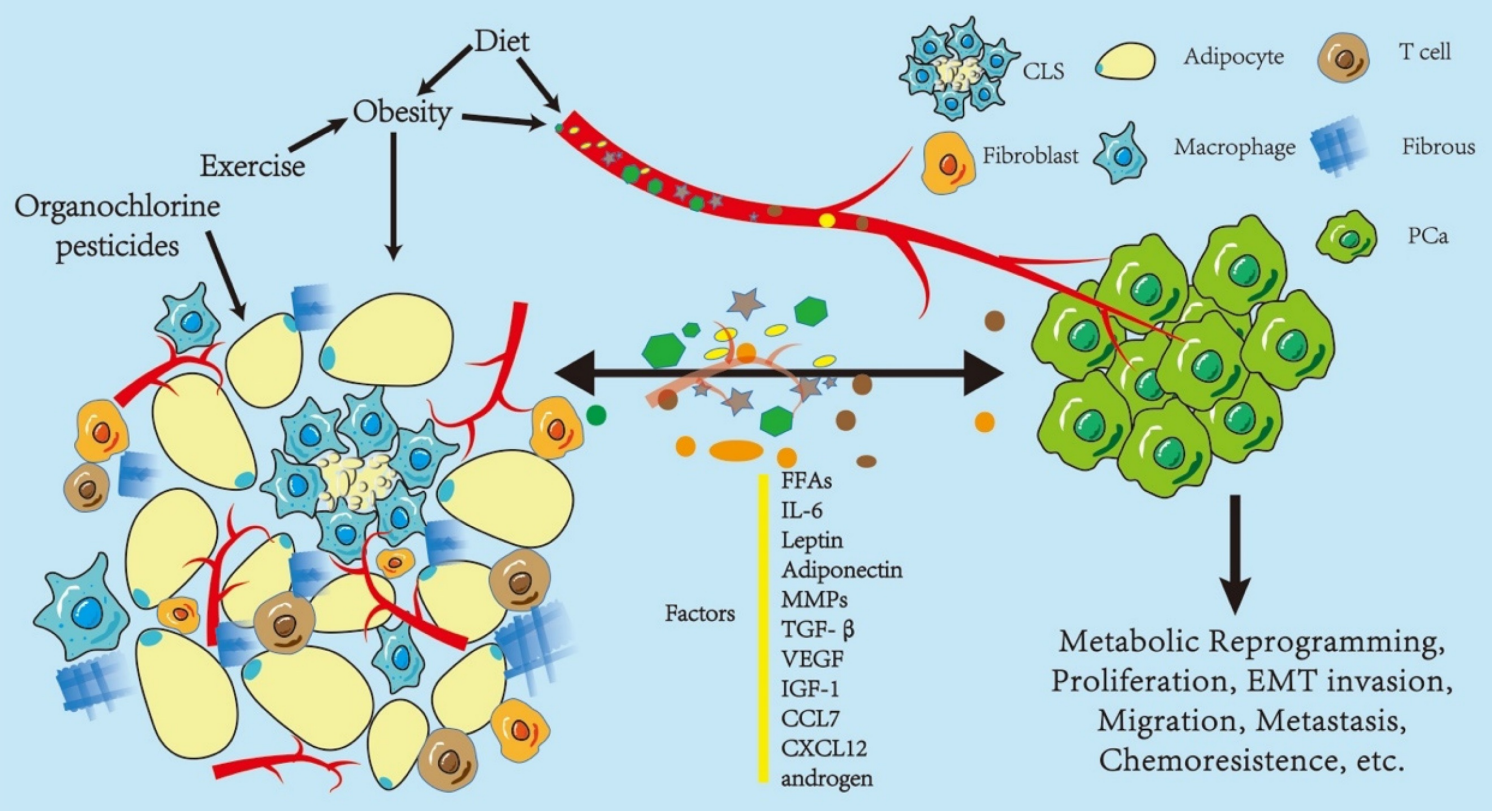
** Influence of periprostatic adipose tissue on prostate cancer.** Periprostatic adipose tissue (PPAT) consists of many adipocytes, other non-adipocytes, connective tissue matrix, blood vessels, and nerve tissues. The non-adipocyte components include inflammatory cells (macrophages), immune cells, preadipocytes, and fibroblasts. These components, as a whole, are capable of secreting various factors that influence the biological behavior of PCa in a paracrine or endocrine manner, including metabolic reprogramming, proliferation, and epithelial-to-mesenchymal transition (EMT) invasion. Some of these factors promote PCa progression, such as IL-6, leptin, VEGF, and CCL7; however, there are protective factors, such as adiponectin, and the effect on PCa depends on the balance between these two kinds of factors. In turn, PCa regulates the biological behavior of adipose tissues, thus promoting its development. Obesity and diet may enhance the effect of PPAT in an endocrine manner. Diet and exercise may indirectly alter PPAT function by affecting obesity or directly change the function of PPAT, and organochlorine pesticide deposition in PPAT may also affect PPAT function. Abbreviations CLS: crown-like structure; PCa: prostate cancer; IL-6: interleukin 6; MMPs: matrix metalloproteinases; TGF-β: transform growth factor-β; VEGF: vascular endothelial growth factor; IGF-1: insulin-like growth factor; CCL7: C-C motif ligand chemokine 7; CXCL 12: C-X-C motif ligand chemokine 12; MCP-1: monocyte chemoattractant protein 1.

**Figure 2:**
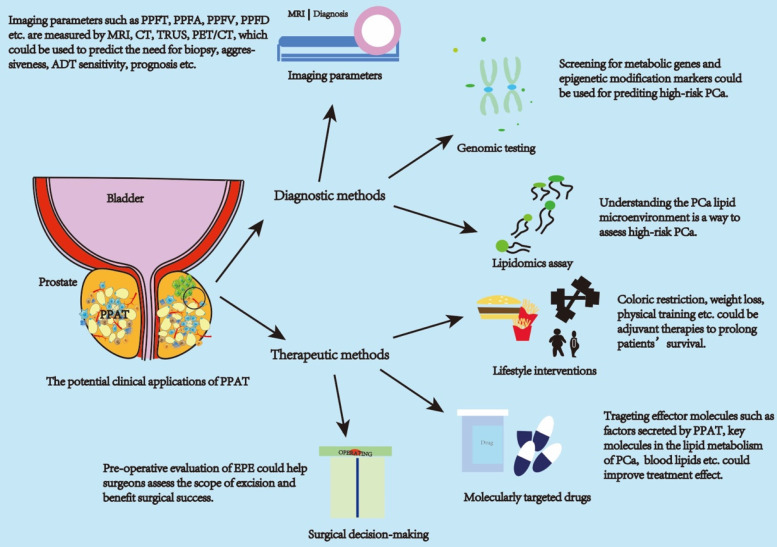
** PPAT has potentially valuable clinical applications.** The clinical applications of PPAT can be divided into diagnostic and therapeutic approaches. Diagnostic approaches focus on applying PPAT imaging parameters, genomics, and lipidomics. Therapeutic approaches can be in molecularly targeted drugs, lifestyle interventions, and surgical approaches to decision-making. Imaging parameters can be used to assess the aggressiveness of PCa, time to CRPC, and patient prognosis. PPAT lipid metabolism genomic and epigenetic assays can be used to predict high-risk PCa. Lipidomic assays can be used to assess the PCa lipid metabolism microenvironment and predict high-risk PCa. Life style intervations and targeted drugs can improve the effect of treatment. The amount and distribution of PPAT can serve as a consideration for the surgeon to predict the surgical plan. Abbreviations: PPAT: periprostatic adipose tissue; PPFT: periprostatic fat thickness; PPFA: periprostatic fat area; PPFD: periprostatic fat density; PPFR: periprostatic fat ratio; PCa: prostate cancer.

Based on a previous study that strongly suggested a strong association between PPAT and PCa (Figure 1), we explored the use of PPAT in clinical diagnosis and treatment (Figure 2).

### 6.1 PPAT for Diagnosing PCa

The above studies illustrate the involvement of PPAT in regulating the biological behavior of PCa through the secretion of various lipid molecules and molecules with effective activity and inflammatory status. This affects the clinical progression of the tumor and is closely associated with patient pathological staging, clinical treatment decisions, biochemical recurrence, survival, and other outcomes. Given this close association, it is crucial to translate PPAT features into clinically accessible measurement parameters for diagnosing patients' disease progression status. Current studies show promising results for diagnosing PCa progression status using PPAT imaging parameters, lipid metabolism-related gene assays, and lipid secretomes assays. PPAT-related imaging studies are the main research directions with promising applications.

The measurement of PPAT imaging parameters is currently the most readily available. Clinicians assess PCa aggressiveness and prognosis by imaging parameters such as periprostatic fat (PPF) thickness (PPFT), PPF area (PPFA), and PPF volume (PPFV) measured using rectal ultrasound (TRUS), computed tomography (CT) and magnetic resonance imaging (MRI). Table 1 summarizes the current imaging clinical studies related to PPAT. MRI is the primary modality for measuring PPAT-related parameters and has advantages over TRUS and CT. MRI has a good resolution of soft tissue and presents clear images. MRI is routinely used for the diagnosis, localization, and risk stratification of patients with PCa and is free of ionizing radiation 84. TRUS is highly operator-dependent, and there may be variability in the choice of the measurement image plane. Moreover, the pressure applied to the prostate during the examination may affect the thickness of the fat surrounding the prostate 85. CT is an excellent differentiator and quantifier of adipose tissues with good density measurements; however, its resolution is poor with an inherent risk of ionizing radiation 84. In addition, some studies have used positron emission tomography (PET)/CT measurements, which are not routinely performed in the initial stages of PCa owing to the lack of additional clinical value for early PCa 86, 87.

Various PPAT parameters, such as PPFT, PPFV, PPFD, and PPFR, were used in the current study, as shown in Table 1. PPFT is the shortest vertical distance from the pubic symphysis to the prostate 84. This method is relatively simple and easy to perform. It is based on only one distance in one plane, and can be performed quickly and reproducibly with basic training to identify relevant anatomical structures on MRI, which may be more suitable for clinical use. However, the method does not reflect the general distribution of PPAT, does not consider the differences in PCa location and fat thickness between the left and right sides, and PPFT is susceptible to the influence of prostate volume and pubic symphysis shape. The results obtained should be carefully validated, and further studies are needed to accumulate more reliable data regarding PCa and PPAT 88. PPFA and PPFV measurements require calculation using advanced imaging software, which is relatively complex and time-consuming in clinical applications, and many hospitals do not have the appropriate equipment 89. In addition, the heterogeneity of PPAT margins, complex weave structure, minute differences from surrounding tissues, inter-patient variability in shape and size, and heterogeneity in intensity distribution makes it difficult to measure the relevant parameters on MRI accurately. A recent study reported an algorithm that can accurately and automatically segment PCa and PPAT in T2-weighted images, reducing clinicians' workload 90. However, further simplification of the measurement modality is still needed to facilitate its clinical application.

Several studies have shown a strong correlation between PPAT and PCa; however, some studies have noted that PPAT is unrelated to PCa 91, 92, 93, which the small sample size and heterogeneous differences in study participants may cause. In addition, when considering PPAT imaging parameters, it may be more accurate to combine the inflammatory status of PPAT 62, Gleason score, PSA level, and Prostate Imaging Reporting and Data System (PI-RADS) to predict the subsequent management of patients with PCa.

In addition to measuring the PPAT imaging parameters, some studies have also diagnosed the risk profile of patients with PCa by measuring the genomic expression of genes in PPAT. The expression was significantly different. Those with low CRTC2 expression had high pGS (pathological Gleason Score) values, high seminal vesicle infiltration, significantly poorer pathological outcomes, and significantly lower biochemical recurrence-free survival 94. Mangiola et al. improved and evaluated the 3-genes (IGHA1, OLFM4, and RERGL) signature in PPAT and obtained discriminatory utility in predicting the presence of high-risk disease 28. By identifying differentially expressed genes with aberrant methylation patterns on PCa, PPAT can differentiate between localized PCa and locally progressive PCa. These genes will be new diagnostic candidate molecular markers 95. In addition, previous studies have reported that the lipid composition of PPAT can, to some extent, indicate the risk of PCa progression. Its detection can help us understand the tumor metabolic microenvironment and provide new stratification factors to assess the PCa risk class 33. Scheinberg et al. also summarized several lipids associated with an increased risk of PCa diagnosis, including 1-stearoyl glycerol, glycerophospholipids, acylcarnitine, and lipids involved in phospholipid metabolism. Furthermore, lipids associated with an increased risk of advanced PCa include phosphatidylcholine and lysophosphatidylcholine, hydroxy sphingosine, or acylcarnitine. Similar trends were observed for aggressive disease and death. However, these studies could not reproduce each other's findings because it is difficult to compare between trials owing to differences in study methods, assays, and metabolites examined 78. Overall, these results suggested that PPAT-related genomic and lipid metabolomic profiles have critical potential applications for diagnostic applications.

### 6.2 Improvement of PCa Treatment Outcome by PPAT

Previous studies have suggested that inhibiting the effects of crucial molecules of PPAT secretome may inhibit PCa progression, such as CXCR4 antagonist AMD3100 inhibiting CXCL12 55, UCB35625 inhibiting the CCR3/CCL7 axis 56, TGF-β receptor inhibiting SB431542 58, and IGF-1 receptor inhibiting AG1024 59. All these inhibitors are molecularly targeted drugs with potential applications requiring further investigation. In addition, several studies have corroborated the potential applications of targeting these molecules. Stoykova et al. suggested that targeting critical metabolic enzymes of PCa lipid uptake, synthesis, and oxidation processes demonstrated anti-PCa effects 79; for example, the FASN inhibitor IPI-9119 improves cancer metabolomics and induces PCa apoptosis, potentially providing a novel approach for preventing and treating metastatic PCa 52. The inhibition of the expression of the fatty acid elongase ELOVL7 also leads to the ablation of CRPC xenograft tumors in mice 114. Carnitine palmitoyltransferase-1 (CPT1A) is an enzyme required for the transport of fatty acyl chains from the cytoplasm to the mito-chondrial membrane gap and subsequent FAO, and the CPT1A inhibitor imodium causes PCa cell growth and decreased AR expression 115. Statins, a class of lipid-lowering drugs used to treat hypercholesterolemia, reduce the risk of advanced PCa 116 and ameliorate the association between high saturated fat intake and increased PCa invasiveness, thereby reducing PCa invasiveness 117. In addition to statins, PCSK9 regulates cholesterol metabolism by attaching to the low-density lipoprotein (LDL) receptor and reducing LDL receptor-mediated removal from circulation 117. Based on the current findings, lipid-targeting agents are unlikely to replace highly effective therapies in metastatic PCa; however, they can be combined to improve patient survival 78.

In addition, other factors secreted by PPAT have been associated with PCa in several studies, and targeting these molecules demonstrated anti-PCa effects. Zhou et al. found that plasma IL-6 and TNF-α levels correlated significantly with graded changes in limited PCa 118 and that the downstream molecule of IL-6, STAT-3 inhibitor Stt, and anti-IL-6R antibody Tcz combined to target tumor cells exhibited anti-cancer effects 119. In addition, IL-6 causes PCa resistance to radiation therapy by upregulating DNA repair-related mole-cules ATM, ATR, BRCA1, and BRCA2 120.

Zhang et al. found that the TGF-β receptor I antagonist alisertib significantly inhibited tumor growth and progression in a TRAMP-C1 cell line-derived subcutaneous tumor model 121. Leptin activation induces PCa cancer proliferation, promote invasion, and inhibit apoptosis 122. Philp et al. further validated that the leptin receptor antagonist Alloaca inhibited LNCaP xenograft tumor growth, delayed progression to CRPC in mice, and suggested that leptin receptor blockade combined with androgen axis inhibition is a promising new therapeutic strategy for treating advanced PCa 123. Hu et al. found that patients with PCa had lower serum lipocalin. The use of the peptide lipocalin receptor (ADIPOR) agonist ADP355 in subcutaneous LNCaP xenograft mice slowed tumor growth and retarded the progression of the serum PCa biomarker PSA 124. This inhibition can be achieved by altering cellular energy, cellular stress, and protein synthesis, ultimately leading to apop-tosis 125. In addition, GV1001 inhibits CRPC cell activity, induces apoptosis, and sup-presses angiogenesis by inhibiting the AKT/NF-κB/VEGF signaling pathway 126. All aspects of PCa progression are closely related to androgen levels and androgen receptor (AR) status. Almost all treatments, from desensitized PCa (CSPC) to deadly resistant PCa (CRPC), target androgen metabolic pathways and AR. Altered androgen metabolism and its response are among the leading causes of drug resistance in PCa 127. These findings further support the potential application of targeting PPAT-secreting molecules to control PCa progression.

Other drugs inhibit PPAT inflammation and may improve the prognosis of male patients with PCa. Pioglitazone, a PPARγ ligand, is used for treating diabetes and has anti-inflammatory properties. Miyazawa et al. found that pioglitazone inhibited PPAT inflammation in obese mice, reduced the density of CLS in periprostatic fat, and inhibited the levels of TNF-α, TGF-β, and chemokine monocyte chemotactic protein-1 (MCP-1), thus improving PCa 69. In addition, supplementation with 17β-estradiol (E2) suppressed caloric intake, induced weight loss, reduced PPAT inflammation in obese mice, and down-regulated the expression of genes associated with inflammation, including Cd68, Mcp1, and Tnf 66. Mangiola et al. suggested that ADT is associated with PPAT pro-inflammation and obesity-like adipose tissue microenvironment. The beneficial effects of ADT treatment may be partially offset by the metabolic and inflammatory side effects of PPAT 128.

In addition to developing relevant drugs, lifestyle interventions such as weight loss, calorie intake control, and increased physical activity may improve the prognosis of patients with PCa, as previously described. Calorie restriction (CR) partially inhibits the progression of PCa by modulating the IGF axis, and IGF-1 receptor (IGF-1R) blockade inhibits PCa xenograft growth. Combining CR with IGF-1R blockade would have a superimposed effect on PCa growth 129. Elliott et al. demonstrated an association between high saturated fat intake and the risk of aggressive PCa 117. In addition, PPAT influences multiple steps of the surgical procedure for radical PCa, and it is associated with surgical difficulty. Preoperative attention to PPAT-related parameters may benefit surgical success 130, but this prognostic factor requires further validation. When PPAT is invaded by PCa can be defined as extraprostatic extension (EPE), which tends to predict a worse prognosis. EPE also influences the doctor's decision on the surgical approach, which may often require more extensive resection 131, 132.

## 7. Conclusions and Future Considerations

The effects of adipose tissues on tumors have long been reported. As different adipose depots in the body have unique morphological structures and physiological functions, further differentiation of the effects of each adipose depot on specific tumors may be beneficial for the precise diagnosis and treatment of tumors. PPAT is only one of the adipose tissues that can secrete these substances in vivo; however, it is the closest adipose tissue to PCa, thus it may play a unique role. The adjacent anatomical location makes PPAT more likely to affect the peripheral zone of the prostate, and whether this is related to the higher incidence of PCa in the peripheral zone requires further investigation. In addition, obesity, expansion of other adipose tissues in the body because of diet, and lipid metabolism directly or indirectly affect PCa, thereby enhancing the effect of PPAT. Controlling these factors may improve the prognosis of patients with PCa. Studies have suggested that PPAT contributes to the development of PCa; however, other studies have suggested that PPAT has little or no effect on PCa 23,72,90,92. Because of the complexity of the mechanisms of action of PPAT on PCa, further studies are needed to explore the mechanisms of PPAT effects on PCa. Furthermore, analysis of the whole genome of PCa PPAT by Ribeiro et al. showed reduced local immune monitoring of PPAT, mainly associated with the down-regulation of complement CFH 133. This suggests that the local immune microenvironment of PPAT can affect PCa; hence, there is scope for further basic research on PPAT. More research evidence is needed to apply PPAT in the clinical setting using specific mechanisms to develop appropriate therapeutic tools in the future. Overall, the study of PPAT provides a new perspective for diagnosing and treating PCa.

## Figures and Tables

**Table 1:** Clinical research related to the association between PPAT and PCa

Study	Country and Year	Patient Number	Method of Measurement	Conclusion
Roermund et al. 91	Netherlands, 2004.04-2008.08	902	SFT/PPFA /PPFD, CT	PPFD is not correlated with PC aggressiveness in patients receving brachytherapy.
Roermund et al. 96	Netherlands, 2003.01-2008.08	932	SFT/PPFA/PPFD, CT	PCa is more aggressive in patients with a higher PPFD.
Bhindi et al. 97	Canada, NA	931	PPFT, TRUS	PPFT can predict high-grade PCa at biopsy.
Allott et al. 18	America, 2005-2011	308	SFT/PPFA/PPFD, CT	Visceral fat is related to more aggressive PCa in patients undergoing radiotherapy.
Tiberi et al. 92	Canada, NA	213	PPFA/SFT, CT	BMI and body fat distribution influence rectal dose. Periprostatic fat is not associated with rectal dose.
Woo et al. 84	Korea, 2013.01-2013.12	190	SFT/PPFT, MRI	PPFT is correlated with pathological Gleason score and can predict high-grade PCa.
Tan et al. 85	America, 2013.08-2015.02	295	PPFT/PPFR, mpMRI	Higher PPFR is significantly related to a more aggressive PCa.
Cao et al. 98	China, 2013.01-2015.12	371	SFT/PPFT,mpMRI	PPFT can predict PCa and HGPCa, paticulary for PCa with PI-RADS grade 3.
Dahran et al. 99	UK, 2010.01-2015.12	162	SFT/PPFV, MRI	PPFV was associated with prostate cancer aggressiveness in patients undergoing RP.
Salji et al. 100	UK, NA	224	PPFV, MRI	The tumor response to ADT is associated with PPFV.
Zhai et al. 101	China, 2013.11-2018.03	56	SFT/PPFR, mpMRI	Periprostatic fat can help predict PCa pathologic upgrading.
Huang et al. 89	China, 2011.06-2017.06	150	PPFT/PPFV, CT or MRI	PPFT predicts the time to CRPC in patients getting ADT.
Di Bella et al. 102	UK, 2005-2011	401	PPFA/PPFD, CT	PPAT increases the risk of recurrence in patients undergoing radiation only but decreases the recurrence risk in patients undergoing radiation and ADT.
Iemura et al. 103	Japan, 2013.03-2017.12	220	SFT/PPFT, mpMRI	PV, Gleason score, and PPFT are independent risk factors for upstaging in men undergoing RP
Lee et al. 104	Korea, 2013.01-2017.12	77	CT-attenuation (HU) and FDG uptake (SUV) of PPAT, PET/CT	PPAT is related to PCa progression.
Sasaki et al. 105	Japan, 2005.03-2014.09	85	SFT/PPFT/PPFR, MRI	PPFT is an independent risk predictor of survival in hormone-naïve patients.
Zhai et al. 106	China, 2016.06-2018.10	660	PPFA /PPFR,MRI	PPFT can help predict PCa or csPCa
Gregg et al. 107	America, NA	175	PPFT/PPFV, MRI	Normalized PPFR was related to shorter progression-free survival.
Zhai et al. 108	China, 2016.06-2018.10	179	PPFA /PPFR, MRI	PPFR can predict lymph node metastasis in patients receving RP.
Chien et al. 109	China, 2009.01-2018.12	60	PPFV, MRI	PPFV was associated with prostate cancer aggressiveness.
Xiong et al. 110	China, 2013.03-2022.05	901	PPFT, MRI	PPFT was related to the detection of PCa and csPCa in PCa biopsy.
Taussky et al. 111	Canada, 2009.03-2016.01	61	PPFD/PPFT, CT	5ARIs appear to affect PPAT volume.
Zhang et al. 112	China, 2006.03-2012.10	184	SFT/PPFT/PPFA, MRI	PPAT can help assess the tumor stage and grade.
Laine-Caroff et al. 93	France,2013.10-2015-03	121	PPFT/PPFV,MRI	PPAT is not associated with PCa aggressiveness.
Shahait, M. et al. 113	America, 2013-2018	98	all surgically resectable visceral adipose tissue anterior to theendopelvic fascia extending from the prostatic base to theapex	PPAT features derived from MRI scans predict patients with clinically significant PCa

PPFT: periprostatic fat thickness; PPFA: periprostatic fat area; PPFD: periprostatic fat density; PPFR: periprostatic fat ratio; PCa: prostate cancer; RP: radical prostatectomy; csPCa: clinical significance PCa.
